# Does improvement towards a normal cervical sagittal configuration aid in the management of cervical myofascial pain syndrome: a 1- year randomized controlled trial

**DOI:** 10.1186/s12891-018-2317-y

**Published:** 2018-11-12

**Authors:** Ibrahim M. Moustafa, Aliaa A. Diab, Fatma Hegazy, Deed E. Harrison

**Affiliations:** 1 Department of Physiotherapy, College of Health Sciences, University of Sharjah, Sharjah, United Arab Emirates; 2 Basic Science Department, Faculty of Physical Therapy, Cairo University, 7 Mohamed Hassan El gamal Street-Abbas El Akaad, Nacer City, Egypt; 3 CBP Nonprofit (a spine research foundation), Eagle, ID USA

**Keywords:** Randomized controlled trial, Cervical lordosis, Cervical posture, Cervical pain, Myofascial pain, Traction

## Abstract

**Background:**

There is a growing interest concerning the understanding of and rehabilitation of the sagittal configuration of the cervical spine as a clinical outcome. However, the literature on the topic specific to conservative treatment outcomes of patients with chronic myofascial cervical pain syndrome (CMCPS) has not adequately addressed the relationship between cervical sagittal alignment and improved pain, disability and range of motion.

**Methods:**

A randomized controlled study with a 1-year follow-up. Here, 120 (76 males) patients with chronic CMCPS and defined cervical sagittal posture abnormalities were randomly assigned to the control or an intervention group. Both groups received the Integrated neuromuscular inhibition technique (INIT); additionally, the intervention group received the denneroll cervical traction device. Alignment outcomes included two measures of sagittal posture: cervical angle (CV), and shoulder angle (SH). Patient relevant outcome measures included: neck pain intensity (NRS), neck disability (NDI), pressure pain thresholds (PPT), cervical range of motion using the CROM. Measures were assessed at three intervals: baseline, 10 weeks, and 1 year after the 10 week follow up.

**Results:**

After 10 weeks of treatment, between group statistical analysis, showed equal improvements for both the intervention and control groups in NRS (*p* = 0.36) and NDI (*p* = 0.09). However, at 10 weeks, there were significant differences between groups favoring the intervention group for PPT (*p*<0.001) and all measures of CROM (*p*<0.001). Additionally, at 10 weeks the sagittal alignment variables showed significant differences favoring the intervention group for CV *p*<0.001 and SH (*p*<0.001) indicating improved CSA. Importantly, at the 1-year follow-up, between group analysis identified a regression back to baseline values for the control group for the non-significant group differences (NRS and NDI) at the 10-week mark. Thus, all variables were significantly different between groups favoring the intervention group at 1-year follow up: NRS (*p*<0.001), NDI (*p*<0.001), PPT *p*<0.001), CROM (*p*<0.001), CV (*p*<0.001), SH (*p*<0.001).

**Conclusion:**

The addition of the denneroll cervical orthotic to a multimodal program positively affected CMCPS outcomes at long term follow up. We speculate the improved sagittal cervical posture alignment outcomes contributed to our findings.

**Trial registration:**

Pan African Clinical Trial Registry Clinical Trial Registry: PACTR201801002968301, registered 11 January 2018 (retrospectively registered).

## Background

Chronic myofascial pain syndrome (CMPS) is a musculoskeletal condition or syndrome that is typically associated myofascial trigger points (MTrP). CMPS has a lifetime prevalence of up to 85% with variations as low as 15% for a point prevalence [1, 2]. CMPS significantly impacts a patient’s health related quality of life outcomes with studies including: disability, financial status, depression, anxiety, and generalized neck pain [3, 4].

Myofascial pain syndrome remains one of the most common sources of pain in chronic non-specific neck pain. Factors commonly cited as predisposing to MPS among subjects with chronic non-specific neck pain include abnormal postural, inadequate rest, overstretching, over-shortening or more generally, repetitive mechanical stress [1, 2]. In clinical practice, different approaches such as massage, acupuncture and electro-thermotherapy are quite commonly used in the treatment of CMPS [3, 4]. However, the effectiveness of many of these approaches did not appear to be superior to placebo [3]. A recent systematic review found that functional exercise protocols have very low quality evidence for a positive small-to-moderate effect on pain intensity in patients suffering from MPS [5].

Identification of causative variables for MTrPs is a first step to prevent development and secondarily to develop potential treatments preventing recurrence. Although the exact mechanisms are still unknown, [6, 7] it is accepted that mechanical factors are thought to be factors associated in the development of MTrPs [1, 2, 8]. In this regard, various studies have confirmed that prolonged abnormal postures have been regarded as one of the causes of MPS [9, 10].

In the cervical region, various studies point to the fact that altered sagittal plane alignment of the cervical spine such as straightened, s-curves, reversed curves, and anterior head translation can result in abnormal stresses and strains leading to premature and acceleration of degenerative changes in the muscles, ligaments, bony structures and neural elements [11–13]. Furthermore, preliminary randomized trials have demonstrated improved neck pain, dizziness, disability, positioning sense, flexion / extension kinematics, arm pain, and somatosensory evoked potentials in patient groups receiving devices aimed at restoration of the cervical curve and posture [14–17]. One such device for the rehabilitation of sagittal cervical alignment is the cervical denneroll spine orthotic out of Sydney, Australia. Two previous clinical trials have demonstrated the denneroll is a reliably placed three-point bending extension traction device that is relatively easy to use by both the patient and treating therapist, and it is effective at improving cervical lordosis (10°-14° improvement) and reducing anterior head translation (10-25 mm reduction) [15, 16].

Although the previously mentioned studies make a significant contribution to understanding the important role of a normal cervical lordotic curve and rehabilitation tools to enhance correction, the literature on the topic specific to conservative treatment outcomes of patients with MPS has not adequately addressed the relationship between cervical sagittal alignment and improved pain and disability at short and at long term follow up.

Accordingly, the present randomized controlled trial was undertaken to investigate the functional and pain response outcomes of denneroll cervical extension traction compared to standard care in patient cases with chronic MPS, with a verified hypo-lordosis and anterior translation of the cervical spine. Two primary hypotheses were tested: 1) that denneroll cervical traction will improve the sagittal alignment of the cervical spine. 2) The secondary hypothesis tested was whether restoration of normal cervical sagittal alignment will improve both short and long-term outcomes in cervical myofascial pain syndrome patients.

## Methods

### Patients

A prospective, investigator-blinded, parallel-group, randomized clinical trial was conducted at one of our university’s research departments, the trial was registered with the Clinical Trial Registry PACTR201801002968301. Cairo university institutional review board approval was obtained prior to the study and all subjects were recruited from our institutions local outpatient clinic. Patients with cervical MPS were recruited from our university’s rehabilitation clinic. Patients were recruited and treated from March 2016 to October 2017 including a 1-year of follow-up.

Participants were screened prior to inclusion for alterations in two primary cervical alignment variables: loss of the cervical lordosis and anterior head translation. As part of our University’s IRB approved protocol, each participant was only to receive initial cervical spine radiography (with no follow up spine radiography) because a primary goal of the cervical denneroll orthotic is to restore the cervical lordosis, thus participants were necessarily screened for hypo-lordosis. Participants were included if their cervical lordosis was less than 25° as measured using the intersection of two lines drawn along the posterior body margins of C2 and C7 [12]. Initial cervical radiological assessment was important to identify the cervical curve apex to determine where a subject should properly place the apex of the denneroll in their cervical spine [16, 18].

Concerning anterior head translation, the participant had to have significant anterior head translation as measured by the craniovertebral angle (CVA). If the CVA was less than 50°, then a participant was referred to the study. Our selection of 50° as a reference angle was guided by the study of Yib et al. [19]. Consecutive patients were included if they had active, palpable MTrPs on a single side or both sides of the upper trapezius muscle. Diagnosis was made according to Travell and Simons’ criteria, whereby five major and at least one minor criteria are required for clinical diagnosis [20]. The major criteria are (1) localized neck pain; (2) pain or altered sensations in expected referred pain area for given trigger point; (3) taut band within the muscle; (4) exquisite tenderness in a point along taut band; (5) restricted range of motion. The minor diagnostic criteria for MPS are (1) reproduction of the patient’s chief pain complaint by manual pressure on MTrP nodule; (2) a local twitch response; and (3) pain relief obtained by muscular treatment. Participants were excluded if any signs or symptoms of medical “red flags” were present: tumor, fracture, rheumatoid arthritis, osteoporosis, and prolonged steroid use. Additionally, subjects were excluded with previous spine surgery and any exam findings consistent with neurological diseases and vascular disorders.

An independent research assistant performed a concealed permuted block randomization using a computer-generated randomization schedule with a random block size.

### Randomization

Our study design randomly assigned eligible participants to 1 of 2 groups: an intervention group (*n* = 60) or control group (n = 60). Examiner blinding was obtained through an independent research assist; not knowing the study design and not specifically involved in any aspect of the trial. This research assistant created a concealed permuted block randomization for subject group allocation; where equal numbers were placed in each group using a permuted block design of different sizes.

### Treatment methods

Both the control group and the intervention groups received the treatment interventions including: Integrated neuromuscular inhibition technique (INIT), Ischemic Compression, Strain Counterstrain (SCS), and muscle energy technique (MET). Additionally, the participants in the intervention group received the denneroll cervical traction. The control group was treated also with a placebo treatment using a small cervical towel applied in the supine position located in the mid cervical spine as an intervention to mimic the denneroll traction time; but without applying significant extension bending of the cervical spine.

Following 30 sessions, participants were re-evaluated a minimum of 24 h after their last session and then each subject was again followed for an additional 1-year time frame with no supervised treatment. The treating therapist (F.H), for both the control and intervention groups, was un-blinded to the treatment method but the subjects and assessor (A.I.M.M. and A.A.D.) who conducted the measurements were blinded.

### Denneroll extension traction for the intervention group

**Fig. 1:**
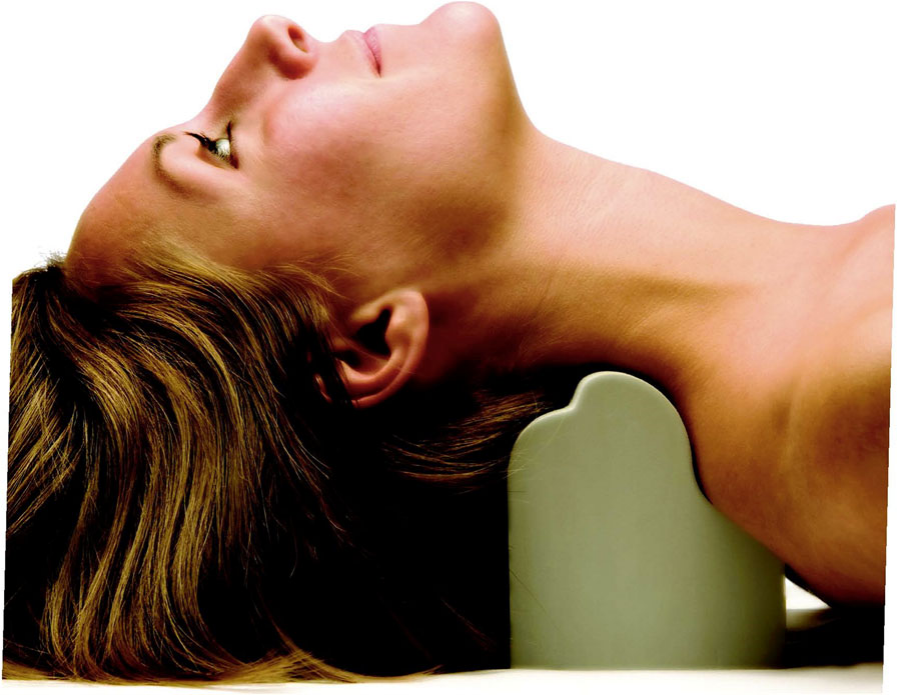
Denneroll cervical spine extension traction. ©Copyright CBP Seminars, reprinted with permission

The participants in the intervention group additionally received the denneroll cervical extension traction (Denneroll Industries, Sydney Australia; http://www.denneroll.com) following previously published protocols [18, 21]. The patients were instructed to lie supine and keep their legs extended. Based on the apex of each participant’s cervical curvature on the initial radiograph, the therapist positioned the apex of the denneroll in one of two regions (mid cervical placement or lower cervical placement). The duration of the traction session started at 2–3 min and increased 1 min per session until reaching the goal of 20 min, the traction was repeated three times per week for 10 weeks. See Fig. 1.

### Integrated neuromuscular inhibition technique (INIT)

The treating therapist first identified the TrPs to be treated within the upper trapezius muscle. The practitioner evaluated the fibers of the upper trapezius, making note of any active TrPs, by firmly pinching using the thumb and the forefinger. Ischemic compression was applied by placing the thumb and index finger over the active TrP. The therapist applied slow, increasing levels of sustained pressure to the area until a relaxation of the tissue barrier was felt. Following a release of pressure, the therapist again applied increased pressure until a new barrier was felt. This process was repeated until the patient indicated the area was no longer tender or until 90 s had elapsed; whichever came first. All identified TrPs were treated in the above manner per generally accepted methodology reported in the literature [6–8].

### Ischemic compression and strain Counterstrain (SCS)

Here, moderate pressure was applied by the therapist to the identified MTrP while each patient rated their level of pain on a scale from 1 to 10. Once the patient’s pain was reproduced, the therapist maintained pressure over the active MTrP and located a position that eased the patient’s perception of pain. This position of ease was generally identified as positioning the affected muscle in a shortened/relaxed state; where a reduction in pain intensity of 70% was indicated by the patient. Once identified, the position of ease was held for 20–30 s and this was repeated for three to five repetitions by the therapist; similar to other generally accepted methodology reported in the literature [6–8].

### Muscle energy technique (MET)

Following SCS, the participants received MET applied to the affected upper trapezius. Here, an isometric contraction was held for 7–10 s and was followed by further cervical spine contralateral side bending, flexion, and ipsilateral rotation to maintain and increase the soft tissue stretch as the muscle belly relaxes. The MET stretch position was repeated three to five times per treatment session and was maintained for 30 s. This protocol is similar to other generally accepted methodology reported in the literature [6–8].

All the participant received the treatment by the same physiotherapist, who had 15 years of experience in manual therapy.

### Home exercise protocol

Participants were advised to perform a home exercise program once daily. The program included strengthening exercises for scapular retractors, deep cervical flexors, and neck extensors. This protocol has been previously reported [18, 21]. The participants were instructed to practice the same home exercise program at least twice a week during the 1 year follow up period. During the follow up period, participants were followed up by telephone interviews every 3 months.

### Outcome measures

The participants underwent a series of assessments at three time intervals: prior to treatment, after 10 weeks of intensive treatment, and at 1 year of follow-up. The order of measurements was the same for all participants.

#### Primary outcome measure

- The Neck Disability Index (NDI), consisting of 10 items related to daily living activities, was our primary patient-reported outcome measure. The reliability, construct validity, and responsiveness to change of the NDI have all been assessed [18, 21].

#### Secondary outcome measures

- Cervical sagittal alignment, neck pain on a numerical rating scale, cervical ROM and pain pressure thresholds via an algometric score were secondary outcome measures.

#### Postural cervical sagittal alignment

**Fig. 2:**
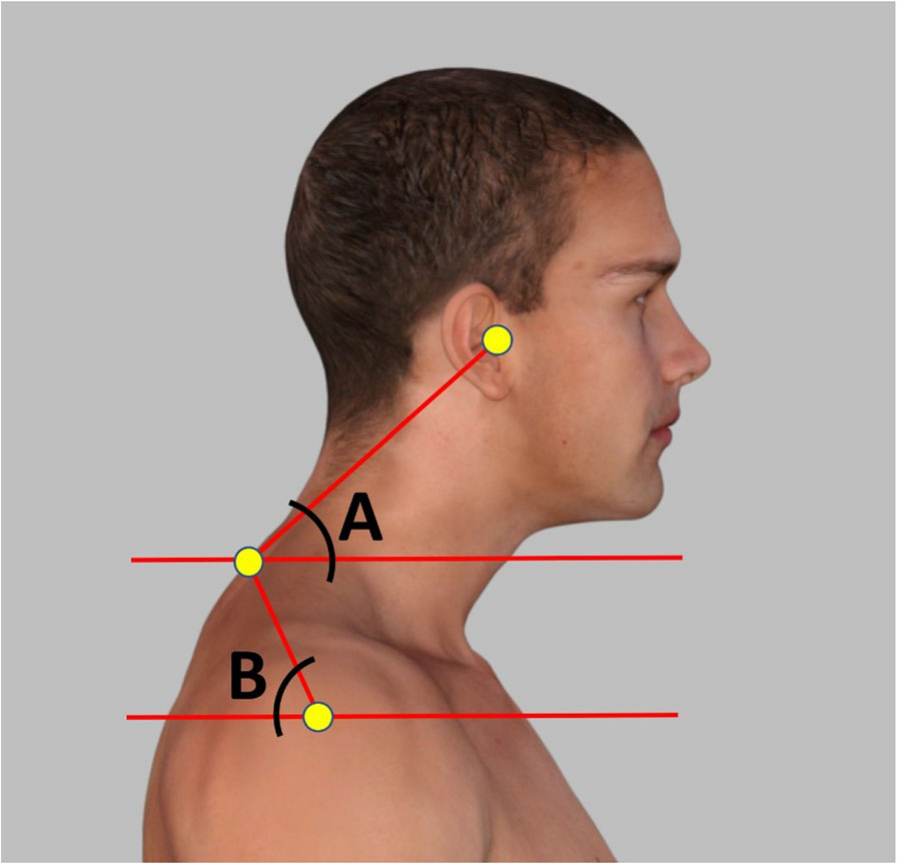
Adhesive marker placement and postural angles used to measure anterior head translation. **a**. cervical angle; **b**. shoulder angle

Standing cervical and shoulder posture was measured with photogrammetry, which provide valid and reliable indicators of the spine [16]. Two angles of measurement were used cervical angle (CV), and shoulder angle (SH) (Fig. 2) - and obtained in the sagittal view as follows:

- **Cervical angle** - The cervical angle is highly reliable to assess forward head translation [17]. It is defined as the angle between the true horizontal line through the spinous process of C7 and a line connecting spinous process of C7 with the tragus. In this study, if the angle was less than 50°, the participant was considered to have forward head posture; where subjects with FHP have a significantly smaller CV when compared with normal subjects [22].
- **Shoulder angle** - A line was drawn between the midpoint of the humerus and spinous process of C7, and the angle of this line to the horizontal line through the midpoint of the humerus was calculated in degrees. In the present study, we considered 52° as the reference angle based on Brink et al. [23].

##### Pain intensity

Neck pain intensity was measured using the numerical pain rating scale (NPRS) [24]. The patients were asked to place a mark along the line indicating their current pain intensity; 0 reflecting “no pain” and 10 reflecting the “worst pain”.

#### Pressure-pain threshold (PPT) algometric measurement

A pressure threshold algometer (Lutron electronic, FG5005, RS232) was used to measure PPT in the most tender point (MTP) of the upper trapezius muscle before and after treatment. The average value of 3 repetitive measurements with an interval of 30 to 60 s (expressed as kilograms per square centimeter) was taken for data analysis of the PPT [25].

#### Cervical ROM

Cervical spine global range-of-motion was measured using the valid and reliable cervical range-of-motion (CROM) device [26]. The participant was instructed to perform flexion, extension, right/left lateral flexion, right/left rotation in upright sitting. The patient was instructed to perform each movement when he/she attained the maximum active range of motion. Three trials were conducted for each direction of movement, and the average of the three measurements were recorded for analysis. All measurements were taken by the same researcher who has postgraduate qualifications and 15 years of clinical experience in musculoskeletal physiotherapy.

### Data analysis

Descriptive statistics were calculated including mean ± standard deviation (SD) for age, height and weight. The outcome measures of NDI, pain intensity, algometric score, CROM, CV angle and SH angle were measured using repeated measures one-way analysis of variances (ANOVA) to compare measurements made before treatment, after the 10 weeks of treatment, and at 1-year follow up. Tukey’s post-hoc multiple comparisons was implemented when necessary. The baseline score for outcomes were used as covariates in a one-way analysis of covariance (ANCOVA) when baseline differences are substantial enough to influence the study outcomes. We considered a mean difference of more than 10.5 points on the NDI as a MCID. Effect sizes measured using Cohen’s d were calculated to examine the average impact of the intervention [27]. According to the method of Cohen, d ≈ 0.2 indicates a small effect and negligible clinical importance, d ≈ 0.5 indicates a medium effect and moderate clinical importance and d ≈ 0.8 indicates a large effect and high clinical importance [24]. For all statistical tests the level of significance was set at *p* < 0.05. Correlations (Pearson’s *r*) were used to examine the relationships between the amount of changes in CV and SH (in the study group) and the amount of change in NDI, pain intensity, ROM, and pressure algometry.

### Sample size

A sample size of 100 patients provided a 90% power of detecting minimal clinically important change (MCIC) on the Neck Disability Index (NDI) of 10.5 points (scale range 0–50. To account for possible participant drop-outs, the sample size was increased by 20% in each group.

Missing values were addressed by using multiple regression models. Model parameters were estimated with multiple regression applied to each imputed data set separately. These estimates and their standard errors were combined into one overall estimate using Rubin’s rules.

## Results

**Fig. 3:**
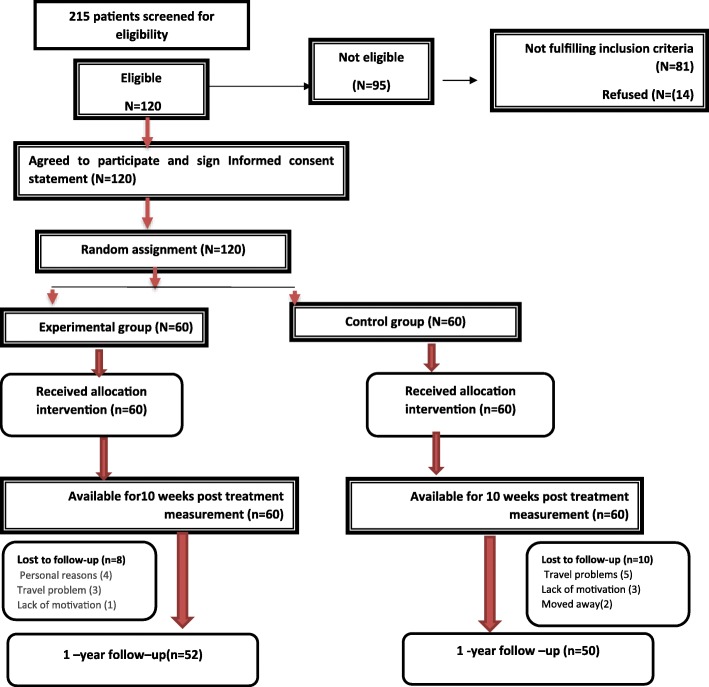
Flow chart of the participants across each part of the study design

A diagram of patient flow and randomization for our study is shown in Fig. 3. Two hundred and fifteen patients were initially screened with 120 of them being eligible to participate in the study. In total 120 (100%) completed the first study follow up after 30 visits or 10 weeks of treatment. At the 1-year follow up, 102 (85%) participants completed the entire study duration. At baseline, both groups were comparable with regard to all variables and had no statistically or clinically relevant differences, except for the cervical rotation ROM and Algometric pressure (Table 1).

**Table 1:** Baseline participant demographics

	Experimental group (*n* = 60)	Control group (*n* = 60)
Age(y)	33.1 ± 8	31.9 ± 7
Weight(kg)	76 ± 10	78 ± 11
Male	40(67%)	36(60%)
Female	20(33%)	24(40%)
Education level		
Primary school	4	3
Secondary school	8	8
Advanced technical colleague	21	20
University diploma	15	13
Others	12	16
Employment status		
Full-time	47	49
Part-time	8	7
Unemployed	5	4
NDI	35.7 ± 2.6	35.1 ± 3.2
Cervical angle	44.8 ± 3.5	45.03 ± 3.1
Shoulder angle	48.2 ± 2.1	48.3 ± 1.7
Pain intensity	5.3 ± .7	5.1 ± .8
Algometric pressure	1.9 ± .2	1.7 ± .3
Cervical flexion	53.2 ± 1	52.8 ± 2.1
Cervical extension	68.1 ± 1.2	67.6 ± 2.5
Right cervical rotation	72.3 ± 2.4	73.7 ± 2.6
Left cervical rotation	72.3 ± 2.4	74.3 ± 2.8
Right lateral flexion	42.3 ± 2	41.9 ± 2.1
Left lateral flexion	42.5 ± 2.1	42.2 ± 2.1

### Primary outcome measure

#### NDI

The difference between the intervention group and the control group was not significant after 10 weeks (*p* = .09; 95% CI [− 1.59 to .131]), however, it was significant at 1-year follow up (< 0.001*; 95% CI [− 11.9–10.23]). The effect size (Cohen’s d) was 0.9 (Table 2). These findings indicated a greater improvement in the interventional group in the NDI and a regression back to baseline-pre-treatment values in the NDI for the control subjects.

**Table 2:** The changes in primary outcomes (NDI) in experimental and control groups vs time

Outcome	Experimental group	Control group	Mean difference (95% CI)	*P* value	effect size (Cohen’s d)	Effect size r
NDI						
Baseline	35.7 ± 2.6	35.1 ± 3.2	[−.5 1.7]	.2		
After 10 weeks	23.3 ± 2.8	24 ± 1.8	[−1.59 .13]	.09		
1-year follow up	17.4 ± 1.3	28.5 ± 1.2	[−11.9-10.2]	< 0.001	8.8	.9
G	< 0.001					
T	< 0.001					
G*T	< 0.001					

*NDI* Neck disability index, G group T: time G vs T: group versus time

### Secondary outcome measures

#### Pain intensity and algometric pressure

Subsequent analyses depending on the presence of interactions for main effects, revealed that after 10 weeks of treatment, the two arms of treatment (both interventional and control groups) seemed roughly equally successful in improving the pain intensity, and pressure algometry outcome measures. At 10 weeks, the unpaired t test analysis revealed insignificant differences between the experimental and control groups for pain intensity (*p* = 0.36), while there was a significant difference for algometry (p<0.001). In contrast, at the 1-year follow-up, the between group analysis showed that there were statistically significant differences in the interventional and control groups for neck pain intensity [0.4 vs. 4.2], *p* < .001), and pressure algometry [3.9 vs. 2], *p* < .001). Table 3 presents this data. The difference between groups at 1-year follow up period reached the MCID for pain intensity and algometric pressure. The effect sizes were 0.67 and .9 respectively (Table 3).

**Table 3:** The changes in secondary outcomes; pain intensity and algometry in experimental and control groups vs time

Outcome	Experimental group	Control group	Mean difference (95% CI)	*P* value	effect size (Cohen’s d)	Effect size r
Pain intensity						
Baseline	5.3 ± .7	5.1 ± .8	[−.05 .5]	.11		
After 10 weeks	1.4 ± .9	1.6 ± .8	[−.5 .17]	.36		
1-year follow up	.4 ± .4	4.2 ± .7	[−4.1-3.6]	< 0.001	.6	.9
G	< 0.001					
T	< 0.001					
G*T	< 0.001					
Algometric pressure						
Baseline	1.9 ± .2	1.71 ± .3	[.15.3]	< 0.001		
After 10 weeks	3.6 ± .3	3.3 ± .5	[.13.5]	< 0.001	.7	.8
1-year follow up	3.9 ± .2	2 ± .4	[1.8 2.1]	< 0.001	.9	.9
G	< 0.001					
T	< 0.001					
G*T	< 0.001					

G: group T: time G vs T: group versus time

#### Cervical angle and shoulder angle

The general linear model with repeated measures indicated significant group x time effects at both the 10 weeks of treatment mark and the 1-year follow up in favor of the interventional group on measures of cervical angle (*p*<0.001) and shoulder angle (*p*<0.001). Table 4 presents group means and standard deviations for each of these variables at each evaluation period. Also, the between group differences with 95% confidence intervals (CI) are presented. The difference between groups after 10 weeks and at 1-year follow up period reached the MCID for all cervical alignment parameters; cervical angle and shoulder angle. The effect sizes on the cervical and shoulder parameters varied from 3.7 to 5.7.

**Table 4:** Changes in secondary outcomes; cervical ROM and posture parameters

Cervical angle (CV)						
	Experimental group	Control group	Mean difference (95% CI)	*P* value	effect size (Cohen’s d)	Effect size r
Baseline	44. 8 ± 3.5	45 ± 3.2	[−1.5 .94]	.6		
After 10 weeks	54.8 ± 2.5	45 ± 3.3	[8.8 10.9]	< 0.001	5.2	.9
1-year follow up	54.2 ± 2.7	44.5 ± 3.1	[8.7 10.8]	< 0.001	5.7	.9
G	< 0.001					
T	< 0.001					
G*T	< 0.001					
Shoulder angle						
Baseline	48.2 ± 2.1	48.3 ± 1.7	[−.77 .6]	.8		
After 10 weeks	57.7 ± 2.9	48.06 ± 1.6	[8.7 10.4]	< 0.001	4.1	.9
1-year follow up	56.9 ± 3.1	48.3 ± 1.4	[7.71 9.4]	< 0.001	3.7	.9
G	< 0.001					
T	< 0.001					
G*T	< 0.001					
Flexion						
Baseline	42.3 ± 2	41.9 ± 2.1	[−.31 1.2]	.2		
After 10 weeks	46.7 ± 1.5	43.3 ± 1.7	[2.8 4.1]	< 0.001	2.5	.7
1-year follow up	46.3 ± 1.4	42.9 ± 2.3	[2.7 4.1]	< 0.001	1.7	.7
G	< 0.001					
T	< 0.001					
G*T	< 0.001					
Extension						
Baseline	68.1 ± 1.2	67.6 ± 2.5	[−.14 1.3]	.1		
After 10 weeks	75.2 ± 1.8	68.2 ± 2.4	[6.3 7.8]	< 0.001	3.2	.9
1-year follow up	74.4 ± 1.7	67.6 ± 1.9	[6.2 7.3]	< 0.001	3.8	.9
G	< 0.001					
T	< 0.001					
G*T	< 0.001					
Right rotation						
Baseline	72.3 ± 2.4	73.7 ± 2.6	[−2.3 -.4]	< 0.001		
After 10 weeks	79.7 ± 1.4	74.8 ± 2.3	[4.2 5.5]	< 0.001	2.6	.8
1-year follow up	78.8 ± .9	74.8 ± 2.1	[3.4 4.6]	< 0.001	2.5	.8
G	< 0.001					
T	< 0.001					
G*T	< 0.001					
Left rotation						
Baseline	72.3 ± 2.4	74.3 ± 2.8	[−2.94-1.05]	< 0.001		
After 10 weeks	79.6 ± 1.4	74.8 ± 2.3	[4.21 5.4]	< 0.001	2.6	.8
1-year follow up	78.8 ± .9	74.8 ± 2.1	[3.4 4.6]	< 0.001	2.5	.8
G	< 0.001					
T	< 0.001					
G*T	< 0.001					
Right tilt						
Baseline	42.3 ± 2	41.9 ± 2.1	[−.31 1.18]	.2		
After 10 weeks	46.7 ± 1.5	43.3 ± 1.7	[2.8 4.05]	< 0.001	2.5	.7
1-year follow up	46.3 ± 1.4	42.9 ± 2.3	[2.724 4.1]	< 0.001	1.7	.7
G	< 0.001					
T	< 0.001					
G*T	< 0.001					
Left Right tilt						
Baseline	42.5 ± 2.1	42.2 ± 2.1	[−.42 1.12]	.3		
After 10 weeks	46.7 ± 1.6	43.45 ± 1.8	[2.6 3.9]	< 0.001	2.5	.7
1-year follow up	46.4 ± 1.4	43.15 ± 2.3	[2.5 3.9]	< 0.001	1.7	.7
G	< 0.001					
T	< 0.001					
G*T	< 0.001					

G: group T: time G vs T: group versus time

#### Cervical range of motion

Similarly, all measures of cervical spine range of motion indicated greater improvement in the interventional group compared to the control group at both the 10-week and 1-year follow up points: flexion (*p*<0.001), extension (*p*<0.001), right rotation (*p*<0.001), left rotation (*p*<0.001), right tilting (*p*<0.001), left tilting (*p*<0.001). Table 4 presents the group means and standard deviations for each of the ROM variables at each evaluation period. Also, the between group differences with 95% confidence interval (CI) values for each variable are presented for the 10 week vs. baseline evaluations and the 1 year follow up vs. baseline evaluations in Table 4. The difference between groups after 10 weeks and at 1-year follow up period reached the MCID for all cervical ROM parameters. The effect sizes varied from 1.7 to 3.8 (Table 4).

### Correlation of posture parameters to primary and secondary outcomes

The amount of change in the CV angle (anterior head translation) at baseline vs. 10 weeks in the intervention group receiving the denneroll significantly correlated with the amount of change in NDI (*p*<0.032), pain intensity (*p*<0.05), pressure algometry (*p*<0.033) and all ROM measures. Whereas, the amount of change in the SH angle at baseline vs. 10 weeks in the intervention group correlated only with pain intensity (*p*<0.015), and algometry (*p*<0.012). The significant correlations were maintained at 1-year follow up with no significant differences in the findings from 10-weeks to 1-year indicating stability. Table 5 presents this data.

**Table 5:** Pearson’s r correlation matrix for outcome variables in the intervention group

	Δ cervical angle 0-10w	Δ cervical angle 10-1Y	Δ shoulder angle 0-10w	Δ shoulder angle 10w -1Y
Δ pain 0-10 W	−.2 P = .05		−.2 P = .015	
Δ pain 10 W-1Y		−.1 P = .2		−.05 P = .3
Δ NDI 0-10w	−.24 P = .032		−.11 P = .196	
Δ NDI 10 w-1Y		.2 P = .0		.07 P = .2
Δ algometric 0–10 w	.24 P = .033		.29 P = .012	
Δ algometric 10w-1 Y		−.027 P = .4		−.002 P = .4
ROM flexion 0–10 w	.2 P = .028		.15 P = .1	
ROM flexion 10w-1 Y		−.16 P = .1		−.007 P = .4
ΔROM extension 0–10 w	.34 P = .003		−.06 P = .3	
ΔROM extension 10w-1 Y		.06 P = .3		.053 P = .3
ΔROM RT rotation 0–10 w	.25 P = .026		.14 P = .1	
ΔROM RT rotation 10w-1 Y		−.11 P = .1		.033 P = .4
ΔROM left rotation 0–10 w	.4 P = .036		.03 P = .4	
ΔROM rotation lt 10w-1 Y		−.1 P = .1		.013 P = .4
ΔROM RT lateral flex 0–10 w	.2 P = .020		.12 P = .1	
ΔROM RT lateral flex 10w-1 Y		.14 P = .1		
ΔROM left lateral flex 0–10 w	.2 P = .021		.05 P = .3	
ΔROM left lateral flex 10w-1 Y		.04 P = .3		−.1 P = .2

## Discussion

Our primary study hypothesis was that reduction or correction of abnormal sagittal cervical posture alignment would impact the short and long-term outcomes of subjects suffering from chronic cervical myofascial pain syndrome (CMPS). Following 30 treatment sessions, our 10-week re-examination findings indicated that there were significant differences between groups favoring the intervention group for sagittal posture alignment, pain pressure thresholds (PPT) and all measures of cervical range of motion (CROM). Further, at the 1-year follow-up, the between group analysis identified a regression back to baseline values for the control group’s neck pain and disability, while the intervention groups variables remained stable where all measures showed statistically significant improvements favoring the intervention group: NRS (*p*<0.001), NDI (*p*<0.001), PPT *p*<0.001), CROM (*p*<0.001), CV (*p*<0.001), SH (*p*<0.001). The above findings confirm our primary study hypothesis.

Regarding improvement in sagittal cervical posture alignment in subjects using the cervical denneroll orthotic, our intervention group’s outcomes are similar to those reported in two earlier trials using this patient prescribed orthotic device [15, 16]. Devices such as the denneroll, act as three-point-bending cervical extension traction devices; where structures located anterior to the axis of extension rotation will be exposed to significant tension loads and structures posterior will experience compression. The anterior tension loading likely unloads the intervertebral disc, causing tension on the anterior cervical spine muscles, and anterior longitudinal ligament, leading to visco-elastic creep deformation resulting in increasing the cervical lordosis and reducing anterior head translation [16, 18, 28].

Anterior head translation and protraction or rounding of the shoulders are likely two postures that are coupled together. In our current study, we identified that the intervention group receiving the cervical denneroll was found to have a reduction in both anterior head posture and a more retracted shoulder / scapular position following treatment. Reduction in anterior head translation is likely responsible for the improvement in shoulder alignment. Similarly, Diab et al., identified that reduction in sagittal head posture was an effective means for improving 3-D spinal posture of the thoracic region and pelvis [29]. Collectively, the finding that rehabilitation of cervical sagittal posture may subtly improve full spine posture measures indicates that there must exist a top down neurophysiological regulation of upright human posture that is driven by the sagittal alignment of the cervical lordosis and forward head posture [30, 31].

### Forward head posture and neck disability index

It is interesting that the application of an integrated neuromuscular inhibition technique alone or in conjunction with an intervention program for forward head posture reduction (denneroll orthotic) seem roughly equally successful in improving neck disability status after 10 weeks of treatment. However, our 1-year follow up data revealed a significant decline in the neck disability index for the control group. The temporal improvement in the control group may be attributed to the strong association between pain relief in both groups and functional status. However, over time, the continuous increased and / or asymmetrical loading from forward head posture may be the possible explanation for the decline in functional disability status for the control group at 12 months follow up. This concept of biomechanical dysfunction resulting from anterior head translation is supported by predictions from experimental and biomechanical spine-posture modeling studies [15, 19, 32, 33] as well as from post-surgical outcomes [34, 35] and large scale cross-sectional investigations [36].

Specifically, Tang et al. [34] identified that anterior translation distance of C2 relative to C7 (termed the SVA) on lateral cervical radiographs positively correlated with the neck disability index in 113 patients receiving posterior cervical spine fusions. Similar results were identified in a prospective sample of 49 patients by Roguski et al. [35]. In a large cross sectional analysis of 656 subjects, Oe et al. [36] identified strong correlations between activities of daily living on the EuroQOL questionnaire and the C2-C7 SVA. These three studies [34–36] are supported by the results of the current investigation where we identified a statistically significant correlation between our experimental groups improvement in their anterior head translation (CV angle) and their consequent improvement in NDI 10-weeks post treatment and at long term follow up.

### Pain intensity and algometric pressure

Overall, our findings revealed a significant and stable decrease in pain intensity for the study group. This long lasting improvement of pain for the study group seems attributable to the restoration of normal posture. It is generally accepted that spinal function is directly related to spinal structure. Abnormal posture elicits abnormal stresses and strains in many structures, including bones, intervertebral discs, facet joints, musculotendinous tissues, and neural elements [13, 27, 32, 33], that can be considered as a predisposing factor for pain from an alteration in mechanical loading. Of interest, in FHP, reciprocal postural compensation was observed in the upper and lower cervical spine to maintain horizontal gaze. FHP caused flexion in the lower segments and extension in the atlanto-occipital and atlantoaxial segments. The transition between flexion and extension occurred in the C2–C4 region. These compensations have implications towards increased abnormal stresses and strains [37]. Thus, restoring the normal sagittal configuration is likely to minimize the abnormal stresses.

This mechanical relationship between prolonged abnormal postures and MPS has previously been identified in different studies [9, 10]. However, few studies have directly evaluated the relationship between forward head posture and MPS in neck and shoulder. Sun et al., examined the correlation between the presence of MPS and abnormal cervical sagittal alignment concluding that “there was no relationship between the forward head position and the presence, location, and number of trigger points” [38]. While Penas et al., highlighted the positive relationship between forward head posture and the presence of active trigger points [39].

The discrepancy and conflict regarding the relationship between abnormal forward head posture with MPS identified by earlier authors cannot be directly compared with our current study because earlier studies are cross-sectional correlation studies without an ability to ascribe cause and effect. In the current study, the significant correlations between the amount of change in the CV angle in the intervention group and neck disability, pain intensity, and algometry outcomes indicates that forward head posture reduction improves the outcomes of MPS.

Concerning the pain level outcomes in the control group, the temporal reduction of pain may be attributed to short term effects of integrated neuromuscular inhibition technique. For example, Hu et al. [40] reported that pain reduction, improvement of MTrP sensitivity, and increase in ROM after various modalities for cervical myofascial pain and trigger-point sensitivity may not be maintained long term. Similarly, the systematic review of Vernon and Schneider [41] provides moderately strong evidence to support the use of some manual therapies in the immediate relief of TrP tenderness. However, only limited evidence to support the use of manual therapies over longer courses of treatments in the management of TrPs and MPS was found.

### Cervical ROM

One might speculate that the improvement of ROM is attributed to a decrease of pain intensity. However, the significant differences between our study and control groups at the two measurement intervals favoring the study group indicate that the loss in ROM is not or not only driven by the presence of myofascial pain [34, 42]. Other factors associated with restricted ROM besides increased muscle tension and pain need considered. Mechanically though, forward head translation alters the anatomic alignment of the cervical spine joints in the sagittal plane, alters the lever arms of the cervical spine muscles and thus this is the most plausible explanation for altered cervical spine ROM [35]. This statement is further supported by the significant correlation between the amount of change in the CV angle in the intervention group and all ROM outcomes. The current study results are logical and agree with those of four other studies [35, 36, 43, 44], each of which investigated the association between forward head and cervical ROM.

### Limitations

The current study has some potential limitations. First, our study lacked blinding of participants and treatment providers. Due to the nature of the interventions, it was not be possible to blind participants and treatment providers to the interventions provided. Second, we used a sample of convenience from 1 clinic; which may not be representative of the entire population of patients with CMCPS. Additionally, we chose selective but relevant patient outcome measures (NDI, pain scale, pain pressure thresholds, range of motion) to identify if changes in sagittal plane posture deviations are related to CMCPS improvement. It is possible that other measures of CMCPS outcomes would have different relationships (greater or less improved) with posture alignment changes and that different interventions than those tested herein may improve patients with CMCPS more considerably.

Third, although the correlations identified between our postural measures and patient outcomes were statistically significant, they would be classified in the moderate range. This indicates that there are other variables, not accounted for in the current study design, which have determining effects on neck disability, pain, and range of motion outcomes. Along this line, we were unable to obtain follow-up lateral cervical radiographs. Thus, we do not if know the cervical lordosis was improved in the experimental group receiving the denneroll and if this may have added any significance to the correlation to patient outcomes.

Within these limitations, the unique contribution of this study is that it evaluated the independent effect of structural rehabilitation of the cervical spine sagittal posture on the short and long term severity of the signs and symptoms associated with CMCPS; which to our knowledge has not been previously reported. A major strength of the present study is the information as to how long pain relief lasted after treatment; up to 1-year. Whereas additional post-treatment measurements with longer than a 1-year interval might have identified a waning effectiveness of treatment.

## Conclusion

This study identified that restoring a more normal cervical sagittal alignment with denneroll traction has a strong positive impact on pain, function, and ROM in patients with myofascial pain syndrome. Our one-year follow-up revealed stable improvement in all measured variables. The findings provide objective evidence that biomechanical dysfunction in terms of abnormal head and cervical posture influences the outcome measures of MPS. These observed effects should be of value to clinicians and health professionals involved in the treatment of MPS where cervical spine alignment rehabilitation can be added to the interventions for MPS patients who present with significant posture abnormality.

## Abbreviations

CMCPS: Chronic myofascial cervical pain syndrome
CV: Cervical angle
INIT: Neuromuscular inhibition technique
NDI: Neck disability index
NRS: Neck pain intensity
PPT: Pressure pain thresholds
SH: Shoulder angle
